# Stimulator of Interferon Genes-Associated Vasculopathy With Onset in Infancy: A Systematic Review of Case Reports

**DOI:** 10.3389/fped.2020.577918

**Published:** 2020-12-17

**Authors:** YunFan Dai, XiuYun Liu, ZhiPeng Zhao, JianXin He, QingQin Yin

**Affiliations:** Department of Respiratory, National Children's Medical Center, China National Clinical Research Center for Respiratory Diseases, Beijing Children's Hospital, Capital Medical University, Beijing, China

**Keywords:** STING-associated vasculopathy with onset in infancy, interstitial lung disease, interferon genes, systematic review, children

## Abstract

**Objective:** To summarize and analyze the manifestations of stimulator of interferon genes (STING)-associated vasculopathy with onset in infancy (SAVI).

**Methods:** A systematic literature review was performed including cases from January 1, 2014, to February 1, 2020, using PubMed, OVID, CNKI, and WanFang. This included all the literature containing comparatively complete clinical data. Statistical analysis was performed using SPSS 20.0 to analyze the difference in age of onset, severity of skin lesions, and respiratory symptoms between SAVI patients with p.N154S and p.V155M mutations.

**Results:** A total of 25 papers were included reporting on 51 individuals, of whom 17 had familiar inheritance of their mutation. Patients included 27 males and 24 females, and 8 fatal cases were observed. A total of 10 mutation sites have been reported in the STING gene, with p.V155M being the most prevalent. We identified SAVI as an early-onset disease with a median age of onset of 3 months after birth. Skin lesions were the most common symptoms of SAVI, found in 94.1% (48/51) of patients, while 76% (19/25) who had undergone a skin biopsy showed vasculopathy. Involvement of the lungs was identified in 68.6% (35/51) of patients, while only 22.2% (4/18) who had undergone a lung biopsy showed vasculopathy. Of 20 patients, 19 had increased immunoglobulin, mainly IgG. Furthermore, 45.1% (23/51) of patients had a positive low titer or were transiently positive for antinuclear antibodies. Of the 18 patients treated with JAK inhibitors, 6 relapsed and 2 died of acute respiratory failure caused by viral infection. Patients with p.N154S mutation had an earlier disease onset (*p* = 0.002) and more severe skin lesions (*p* < 0.001) than those patients with p.V155M mutation.

**Conclusion:** SAVI is an early-onset disease accompanied by skin and lung lesions whose clinical presentation varies among patients with different genotypes. Therapeutic effects of JAK inhibitors are unsatisfactory.

## Introduction

Stimulator of interferon genes (STING)-associated vasculopathy with onset in infancy (SAVI), first reported in 2014 (1), is an interferonopathy caused by gain-of-function mutations in *STING1* gene. It usually involves skin and pulmonary lesions, accompanied by systematic inflammatory symptoms such as a recurrent fever (1). However, the initial manifestations and therapeutic effectiveness differ across reported cases. Here, we conducted a systematic review of all reported SAVI cases, summarizing the characteristics of disease presentation and provide clinical support for early diagnosis and prognosis.

## Materials and Methods

**Figure 1:**
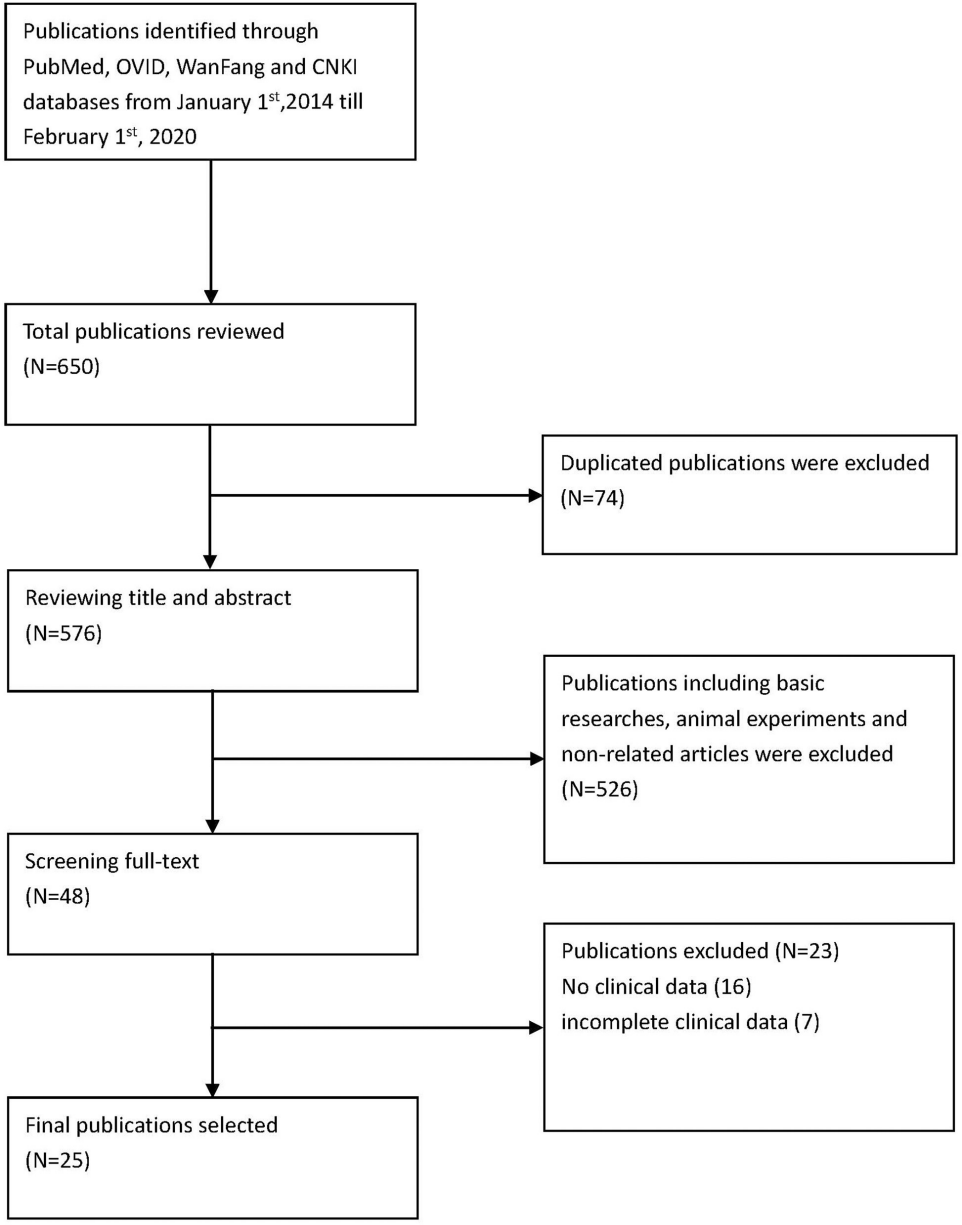
Flow chart of the studies identified in the systematic review.

Methods are summarized in Figure 1.

### Search Strategy

*Via* PubMed, OVID, CNKI, and WanFang, the English terms searched were “sting-associated vasculopathy with onset in infancy” and “stimulator of interferon genes associated vasculopathy with onset in infancy,” “STING,” “TMEM173,” and “mutation.” We searched for literature published from January 1, 2014, to February 1, 2020 (Figure 1). The specific search queries utilized are listed below.

#### PubMed

Formula 1: [(STING-associated vasculopathy with onset in infancy) OR stimulator of interferon genes associated vasculopathy with onset in infancy] AND 2014/01/01:2020/02/01[dp]

Formula 2: {[(stimulator of interferon genes) OR TMEM173] AND mutation} AND 2014/01/01:2020/02/01[dp]

#### OVID

Formula 1: (STING-associated vasculopathy with onset in infancy) OR (stimulator of interferon genes associated vasculopathy with onset in infancy)

Formula 2: [(stimulator of interferon genes OR TMEM173) AND mutation]

We also searched CNKI and WanFang Database for literature published in Chinese using a similar search strategy.

**Inclusion criteria:** (1) including case reports, (2) complete clinical data, (3) articles written in English and Chinese.

## Results

### Summary of Patients in the Included Literature

We included 25 articles (1–25) that met the search criteria, 23 in English and 2 in Chinese. These articles comprised 43 non-fatal and 8 fatal cases, with a sex ratio of 1.25:1 (27 males to 24 females). Moreover, there were 17 familial cases with autosomal dominant inheritance. A total of 10 mutation sites have been reported, with p.V155M being the most prevalent (Table 1).

**Table 1:** Summary of the literature regarding STING1 mutations.

**Authors, Year**	**Cases**	**Mutation sites**	**Familial (F)/sporadic (S)**	**Immunosuppressants**
Liu et al. (1)	6	N154S (4) V155M(1) V147L(1)	S	Hydroxychloroquine, azathioprine, leflunomide, methotrexate, cyclosporine, cyclophosphamide, colchicine, thalidomide, rituximab, tocilizumab, infliximab, etanercept, and mycophenolate mofetil
Jeremiah et al. (2)	4	V155M(4)	F	Methotrexate, mycophenolate mofetil, anti-TNF monoclonal antibody, and anti-CD20 monoclonal antibody
Caorsi et al. (3)	1	V155M	S	Azathioprine and etanercept
Munoz et al. (4)	1	V147M	S	Mycophenolate mofetil, colchicine, hydroxychloroquine and methotrexate, rituximab
Chia et al. (5)	1	N154S	S	Azathioprine
Clarke et al. (6)	1	V155M	S	Not mentioned
Fremond et al. (7)^*^	1	V155M	S	Not mentioned
Picard et al. (8)	3	V155M	F(2) S(1)	Hydroxychloroquine
König et al. (9)	5	G166E	F	Not mentioned
Manoussakis et al. (10)	1	C206Y	S	Not mentioned
Melki et al. (11)	3	R281Q(1) R284G(1) C206Y(1)	S	Methotrexate and anti-TNF-α monoclonal antibody
Dagher et al. (12)	1	V155M	S	Azathioprine
Seo et al. (13)	1	S102P+ F279L	S	Not mentioned
Gallagher et al. (14)	1	C206Y	S	Methotrexate and mycophenolate mofetil
Saldanha et al. (15)	1	R284S	S	Not mentioned
Yu et al. (16)	1	V155M	S	Not mentioned
Cao and Jiang (17)	2	V155M(1) N154S(1)	S	Not mentioned
Keskitalo et al. (18)	6	G207E	F	Methotrexate, azathioprine, and cyclosporine
Shoman et al. (19)	1	N154S	S	Methotrexate
Volpi et al. (20)	3	V155M(1) R281Q(1) N154S(1)	S	Azathioprine, methotrexate, infliximab and etanercept
Zhang et al. (21)	1	V155M	S	Not mentioned
Abid et al. (22)	1	V147L	S	Not mentioned
Balci et al. (23)	1	N154S	S	Not mentioned
Carmela Gerarda Luana et al. (24)	1	V155M	S	Methotrexate and rituximab
Tang et al. (25)	3	V155M	S	Cyclophosphamide

* *Fremond et al. (7) included clinical data of three patients; two of these had been previously reported and supplemented the therapeutic effect of JAK inhibitors, so only one new case was included in this study*.

### Manifestations of SAVI Patients

Our systematic review of the literature found that the age of onset of SAVI ranged from neonatal to adulthood, while the median age of presentation was 3 months after birth (Table 2). The initial symptoms of 35.3% (18/51) of patients were respiratory symptoms, such as tachypnea, dyspnea, cough, and milk-choking, while 56.86% (29/51) initially suffered from skin lesions, accompanied by growth retardation (Table 3). As the most typical skin lesions, chilblain lesions were usually cold-related, including telangiectatic, pustular, erythema, blistering rashes, and ulcers, predominantly on the cheek, auricle, and extremities. Aggravation of the skin lesions resulted in tissue loss, such as nasal septum perforation (12 patients), nail loss (11 patients), and gangrene of the extremities or amputation (13 patients). A total of 35 patients had lung involvements. Among them, 30 cases suffered from respiratory symptoms and 34 cases had signs of interstitial lung disease (ILD) on chest image. Five patients without respiratory symptoms had ILD signs on chest image, while one patient without ILD image change had respiratory symptoms. Other accompanying symptoms included a recurrent fever (30 patients), growth retardation (33 patients), myositis/myalgia (5 patients), arthritis/arthralgia (15 patients), renal impairment (3 patients), and brain impairment (3 patients).

**Table 2:** Clinical characteristics of the 51 SAVI patients.

**Characteristics**	**Cases**	**Percentage**
**Sex**		
Male	27	52.9%
Female	24	47.1%
**Age of onset**		
Under 1 month	17	33.3%
1 month to 1 year	21	41.2%
Over 1 year	12	23.5%
Not mentioned	1	2%
**Skin involvement**	35	68.6%
Recurrent skin lesions	35	68.6%
Nasal septum perforation	12	23.5%
Extremities gangrene/amputation	13	25.5%
**Lung involvement**	35	68.6%
Cough	30	58.8%
Tachypnea/Dyspnea	25	49.0%
Hypoxia	12	23.5%
Interstitial lung disease	34	66.7%
Abnormal lung function	17/21	80.9%
**Systematic inflammation**		
Increased CRP/ESR	32	62.7%
Recurrent fever	30	58.8%
Myositis/myalgia	5	9.8%
Arthritis/arthralgia	15	29.4%
**Antibodies**		
ANA	23	45.1%
ANCA	12	23.5%
**Clubbed fingers**	11	21.6%
**Growth retardation**	33	64.7%
Histological^*^		
Vasculopathy on lung biopsy	4/18	22.2%
Vasculopathy on skin biopsy	19/25	76.0%
**Treatment**		
Corticosteroid	30	58.8%
Immunosuppressant	23	45.1%
JAK inhibitors	18	35.3%

* *Of all 51 patients, 18 underwent lung biopsy (including one who underwent autopsy) and 25 underwent skin biopsy*.

**Table 3:** Initial symptoms of the different genotypes.

		**Initial symptoms**				**Total**
		**Skin symptoms**	**Lung symptoms**	Growth retardation^*^	**Not mentioned**	
Mutation sites	p.N154S	6	3	0	0	9
	p.V155M	5	11	2	2	20
	p.C206Y	3	0	0	0	3
	p.G207E	6	0	0	0	6
	p.G166E	5	0	0	0	5
	p.R281Q	0	2	0	0	2
	p.V147L	2	0	0	0	2
	p.R284G	1	0	0	0	1
	p.R284S	0	1	0	0	1
	p.S102P+F279L	0	1	0	0	1
	p.V147M	1	0	0	0	1
Total		29	18	2	2	51

* *Only two patients presenting without skin or lung symptom are included, and the remaining five patients are grouped into the relevant categories referring to when the skin or lung symptoms were noticed with growth retardation simultaneously*.

### Analysis of Patient Test Data

The inflammatory markers, C-reactive protein, and erythrocyte sedimentation rate were elevated in 62.7% (32/51) of patients. Of the 20 patients who underwent immune function testing, 19 had hyperimmunoglobulinemia, mainly IgG (18/19 patients) (Table 2). The lymphocyte profile was abnormal in 12/18 of patients, with 8 showing decreased CD4+ T lymphocytes and 3 showing elevated CD19+ B lymphocytes. Twenty-three patients presented with a low titer or transiently positive antinuclear antibodies (with the maximum titer of 1:640), and 12 had positive or transient positive anti-neutrophil cytoplasmic antibodies. A total of 10 patients were positive for rheumatoid factor (RF); because 60% (6/10) of patients suffered with arthralgia, we suspected that RF presence and arthralgia symptoms were interconnected. Other related autoantibodies in the patient data included anti-double-stranded DNA antibody (two patients), antiphospholipid antibodies (six patients), anti-cyclic citrullinated peptide antibody (two patients), and lupus anticoagulant (one patient). Over half of the patients (34/51) had signs of ILD on chest high-resolution computed tomography (HRCT), presenting as ground glass opacity, cysts, reticulations, interlobular septal thickening, or pleural thickening. Additional symptoms may also appear in SAVI patients, including consolidations, bronchiectasis, emphysema, lymphadenopathy, and pulmonary hypertension. Only one patient with the p.S102P+F279L mutation presented with indicated obliterans bronchitis. Meanwhile, the familial p.G166E and p.G207E cases had neither respiratory symptoms nor pulmonary lesions on HRCT. Skin biopsies were performed on 25 patients, revealing that 76.0% (19/25) of them had vasculopathy, including vasculitis (13 patients) and perivascular inflammation (6 patients). Another two patients presented with nodular granulomatous dermatitis. All the eight patients who did skin biopsies, with p.N154S mutation, presented vasculopathy, whereas only 4 of 18 patients showed vasculopathy upon pulmonary biopsy in patients with p.V155M mutation.

### Treatment and Prognosis

Previous studies indicated that corticosteroid and multiple immunosuppressive therapies were not effective (1, 21). Among the 51 patients, 30 were treated with corticosteroids and 23 were treated with immunosuppressants, including hydroxychloroquine, mycophenolate mofetil, azathioprine, leflunomide, methotrexate, cyclosporine, cyclophosphamide, colchicine, thalidomide, rituximab, tocilizumab, infliximab, and etanercept. Only 10 patients showed a partial or transient improvement with these therapies; however, in 3 patients, treatment with steroids combined with immunosuppressants (azathioprine, cyclosporine, and methotrexate) stabilized their condition. Since IFN-β stimulates downstream inflammation mainly through the JAK-STAT pathway, JAK inhibitors (tofacitinib, ruxolitinib, and baricitinib) were prescribed to 18 patients. Although 11 patients showed improvement in both skin and respiratory symptoms and 7 patients benefited from partially symptom relief, 6 patients relapsed and 2 patients died of acute respiratory failure caused by viral infection.

### Disease Manifestations Differ Based on Genotype

When summarizing the manifestations of SAVI, the different clinical phenotypes were noticeable. Therefore, the features of patients with p.N154S and p.V155M mutations were compared, including the age of onset and respiratory and skin symptoms. A classification of each patient was made to evaluate the severity of their symptoms (Table 4). A two-samples rank sum tests was used in SPSS20.0 to determine any statistical significance between the genotypes. The results were as follows: patients with p.N154S mutation had an earlier disease onset (*p* = 0.002) and more severe skin lesions (*p* < 0.001) than those patients with p.V155M mutation, whereas there was no difference in respiratory symptom presentation (Table 5).

**Table 4:** Severity grades of p.N154S and p.V155M.

**Lung involvement**	**Grades**	**Number of cases**		**Skin involvement**	**Grades**	**Number of cases**	
		**N154S**	**V155M**			**N154S**	**V155M**
No respiratory symptoms	0	2	4	No skin symptoms	0	0	4
Cough, milk-choking, but no tachypnea/dyspnea	1	1	1	Recurrent rashes, telangiectasia, pustules, blister rash, but no necrotic ulcer	1	1	12
Tachypnea/dyspnea	2	3	7	Necrotic ulcers	2	2	3
Hypoxia	3	1	4	Gangrene amputation or nasal-septum perforation	3	6	1
Die of pulmonary complications	4	2	4				

**Table 5:** Clinical comparison between p.N154S and p.V155M mutations.

	**Rank mean**		***Z***	***P***
	**N154S**	**V155M**		
Onset age^*^	7.39	17.87	−3.159	0.002
Skin lesions	23.00	11.40	−3.599	<0.001
Lung lesions	14.33	15.30	−0.292	0.799

* *The age of onset in one patient with p.V155M mutation did not have a definite value, so it was removed*.

## Discussion

Several mutations have been identified in the *STING1* gene, which encodes a transmembrane protein localizing to the endoplasmic reticulum that is composed of 379 amino acids. STING is a significant signal transduction molecule in innate immunity with important antiviral roles and has functions in cancer (26). It stimulates type I IFN-mediated pro-inflammatory cytokines through the interferon regulatory factor 3 or nuclear Factor kappa B pathways after identifying exogenous ds-DNA or RNA in the cytoplasm (26–33). Various cell types express STING, including endothelial cells, skin cells, hematopoietic cells, T cells, macrophages, dendritic cells, type 2 bronchial epithelial cells, and alveolar cells (1). Therefore, multiple tissues, such as the skin vasculature and pulmonary system, are likely affected by the various mutations, thus resulting in several phenotypes and disease manifestations. The literature describes SAVI as an early-onset disease, with chilblain lesions, ILD, and recurrent fever as its features (1). Our systematic review of the literature showed that the median age of onset in our study was 3 months after birth, with 64.7% (33/51) of patients suffering from both skin and respiratory lesions. However, the spectrum of disease manifestations continues to grow as more genotypes related to SAVI are discovered, including arthritis, myositis, kidney damage, brain damage, photosensitivity, hair loss, and thyroid damage (2, 3, 8, 9, 11, 18, 20, 22, 25). In support of this, 3 cases without skin lesion (21, 25) and 16 cases without pulmonary impairment (1, 2, 11, 18) have been reported, suggesting the clinical phenotypes of SAVI are more diverse than was previously thought.

As hotspot mutations, clinical manifestations of p.N154S and p.V155M were compared, including age of onset, skin, and respiratory symptoms. Compared to patients with p.V155M mutation, p.N154S mutations had an earlier onset and more severe skin lesions. Similar heterogeneity on phenotypes was observed in mouse models. Motwani et al. (34) confirmed that only the V154M mice developed lung fibrosis and the V154M mutant was more active than the N153S mutant inducing four-fold greater levels of IFN-β reporter gene in transfected 293T cells. Meanwhile, lung impairment was not involved in all members of the p.G166E and p.G207E family cases (9, 18). Besides, p.G207E families' patients presented with livedo reticularis and suppurative necrosis of the skin rather than typical chilblain lesions. These families also presented with peculiar features, including light sensitivity, hair loss, abnormal thyroid function, and recurrent sinusitis (9, 18). Above all, we conclude that the clinical phenotypes vary in relation to the different genotypes of SAVI.

Previous research has indicated that SAVI is refractory to corticosteroid and multiple immunosuppressants, while JAK inhibitors may have a curative effect via decreasing the expression of IFN and downstream pro-inflammatory factors. However, when summarizing the 18 patients who had been treated with JAK inhibitors, we found the treatment to be unsatisfactory. Although most JAK inhibitor-treated patients had achieved total or partial transient relief of symptoms, there were six patients who relapsed and two patients who died due to pulmonary complications. The poor therapeutic effect may be explained by the fact found in mouse models that the STING-associated disease depended on T cells but not type I interferon or IRF3 (Interferon Regulating Factor 3) (34–37). Besides, the risk of viral infection might increase in patients treated with JAK inhibitors (5, 7), as infection has been shown to aggravate pulmonary fibrosis in rats (38). Moreover, Saldanha et al. (15) reported on a patient who did not suffer any serious pulmonary infection for 2 years while on immunoglobulin therapy and sulfamethoxazole to prevent infection. Therefore, it can be speculated that preventing infection may delay the aggravation of pulmonary fibrosis and reduce the occurrence of JAK inhibitor side effects. In addition, nitro fatty acids and nitrofuran are direct inhibitors of the STING pathway and could be used to treat STING-related autoimmune diseases in the future (39, 40).

## Conclusion

The clinical phenotypes of SAVI are diverse and related to the genotypes. Compared to the patients with p.V155M mutation, the onset of p.N154S mutation was earlier, with skin lesions of greater severity. The efficacy of JAK inhibitors leaves something to be desired based on previous reports, and our data presented here. Using antibiotics plus immunotherapy to prevent infection may improve patient prognosis.

## Data Availability Statement

The original contributions presented in the study are included in the article/supplementary materials, further inquiries can be directed to the corresponding author/s.

## Author Contributions

YD acquired and analyzed the data and wrote the manuscript draft. XL contributed to design the search criteria and summarized the conclusion. ZZ, JH, and QY made critical revisions to the manuscript. All authors reviewed the manuscript and completed a final approval.

## Conflict of Interest

The authors declare that the research was conducted in the absence of any commercial or financial relationships that could be construed as a potential conflict of interest.
